# Selenium Levels and Antioxidant Activity in Critically Ill Patients with Systemic Inflammatory Response Syndrome

**DOI:** 10.3390/metabo12040274

**Published:** 2022-03-22

**Authors:** Lourdes Herrera-Quintana, Héctor Vázquez-Lorente, Jorge Molina-López, Yenifer Gamarra-Morales, Elena Planells

**Affiliations:** 1 Department of Physiology, Faculty of Pharmacy, Institute of Nutrition and Food Technology “José Mataix”, University of Granada, 18071 Granada, Spain; lourdesherrera@ugr.es (L.H.-Q.); hectorvazquez@ugr.es (H.V.-L.); jennifer_gamo@hotmail.com (Y.G.-M.); 2 Faculty of Education, Psychology and Sports Sciences, University of Huelva, 21007 Huelva, Spain

**Keywords:** selenium, antioxidant, severity, critically ill patient, intensive care unit, systemic inflammatory response syndrome

## Abstract

The Selenium (Se) status could be an important modifiable factor in critically ill patient outcomes due to the important role of this mineral in several functions. Although there are many clinical trials with Se interventions in the literature, the evidence is not sufficient to establish a common criterion regarding the Se status. Background and aims: An analysis was made of the evolution of selenium (Se) and antioxidant status in critically ill patients with Systemic Inflammatory Response Syndrome (SIRS) over 7 days of staying in the Intensive Care Unit (ICU). Methods: A prospective analytical study was carried out on 65 critically ill patients aged 31–77 years. A healthy control group of 56 volunteers from the same region was recruited to allow comparisons with reference normal values. The selenium levels in both the plasma and erythrocytes were analyzed by Inductively Coupled Plasma Mass Spectrometry (ICP-MS). Glutathione Peroxidase (GPx) and Superoxide Dismutase (SOD) activity and the Total Antioxidant Capacity (TAC) were measured using kinetic colorimetric methods. Results: Low erythrocyte and plasma Se levels were found at ICU admission in comparison with the healthy reference group (*p* < 0.001), and the levels further decreased after one week (*p* < 0.001). Smaller changes in the plasma Se levels were associated with greater changes in the Sequential Organ Failure Assessment (SOFA) score (*p* < 0.05). The GPx activity in the critically ill was lower than in the control group (*p* < 0.05), with an inverse correlation to the severity scores at the baseline (*p* < 0.05) and reaching normal values after one week (*p* < 0.05). SOD activity was directly correlated to TAC (*p* = 0.03), with both parameters exhibiting a direct correlation to albumin (*p* < 0.05) after 7 days of ICU stay. Conclusions: A deficient Se status was observed at ICU admission and worsened further over follow-up regardless of the evolution of the patient severity and the antioxidant parameters. Adequate Se support from the start of admission could preserve and contribute to improve the Se-related outcomes and critical patient recovery during longer periods in the ICU.

## 1. Introduction

Critically ill patients constitute a heterogeneous population characterized by the presence of life-threatening multisystemic disorders [1]. These patients are exposed to different stressors that may lead to acute stress and inflammation, which often are associated with a range of poorly understood metabolic disturbances [2]. When this inflammatory condition is not amended, it may generate Systemic Inflammatory Response Syndrome (SIRS), defined as an exaggerated host defense response to a noxious stressor, with the dysregulation of proinflammatory and anti-inflammatory pathway homeostasis [3].

Decreased concentrations of trace elements, especially in the plasma or serum, are a common finding in this population as a result of the inflammatory condition [4]. The micronutrient status plays an important role in maintaining the homeostasis and could prove the determinant in patient recovery. However, it is not clear whether these changes in mineral homeostasis are beneficial or detrimental for critically ill patients [5]. In particular, for Selenium (Se), recent studies have reported decreased Se levels in critically ill patients as early as Intensive Care Unit (ICU) admission compared to patient controls, showing that these values remained stable and were not associated with severity scores or inflammatory parameters such as Sequential Organ Failure Assessment (SOFA) and C-Reactive Protein (CRP) [6]. Contrary, other researchers would have reported the relationship between low Se levels and patient clinical severity according to the simplified acute physiology score and sepsis diagnosis [7], also the strongest predictive factor for ICU mortality [8] Furthermore, the plasma Se status could be affected differently by the patient’s inflammation, finding that erythrocyte Se would not be affected by the inflammatory response [9]. Given the discrepancies in the literature, it is necessary to elucidate the relationship that Se could have with the patient’s clinical outcomes and follow-up that may affect their mortality. Moreover, this complex situation in critical illness influences the metabolism and nutritional requirements, with nutrient supply being a possible modifiable risk factor for patient outcomes [10,11].

In this line, trace elements such as Se have been regarded as potential key pharmaconutrients due to their immunological and antioxidant functions [12]. In this regard, Se is incorporated in selenoproteins, with Glutathione Peroxidase (GPx) being the major component of the mammalian antioxidant defense system within the selenoproteome [13]. Se and selenoproteins participate in a wide variety of processes, and their role in maintaining the erythrocyte redox homeostasis is one of their most remarkable functions [14]. In a critical illness, the Se metabolism could be altered by stress and inflammation, suffering redistribution processes. Due to this situation, low Se levels do not necessarily indicate deficiency, making the definition of reliable reference values difficult [15]. In regard to this, it has been carried out in different clinical trials with Se supplementation, even using high doses [16]. However, recent reviews and metanalyses, which included randomized controlled trials, concluded that the quality of evidence is very low and the risk of bias very high [17,18]. Many factors could influence the potential therapeutic use of Se, such as the previous Se status, which is related to the region [19].

Thus, based on the abovementioned aspects, Se is seen to play a key role in the antioxidant defense, which could be crucial for the recovery of critically ill patients. However, there is not enough evidence to allow us to establish a common criterion regarding the Se status in this population. The aim of the present study was to assess the evolution of both the Se and antioxidant levels in Spanish critically ill patients with SIRS over 7 days of stay in the ICU, as well as the adequacy of Se support. We hypothesized that Se is decreased both at the baseline and after 7 days of ICU stay, influencing both the severity and antioxidant activity of the studied individuals.

## 2. Results

The mean differences in the clinical characteristics between the patients upon admission to the ICU and the controls are represented in Table 1. The mortality rate after 7 days of ICU stay was over one-third of the total study population. Erythrocyte Se expressed per hemoglobin and SOD activity showed no differences between the groups (*p* > 0.05 in all cases), whereas the rest of the studied parameters changed significantly on comparing the ICU patients versus the healthy controls (*p* < 0.001 in all cases).

Table 2 shows the differences in the main outcomes of the study after 7 days of ICU stay. Regarding the plasma and erythrocyte Se, which were below the reference values at the time of ICU admission, the levels were seen to further decrease after 7 days of ICU stay (*p* < 0.001). In particular, the prevalence of individuals with a high risk of Se deficiency increased after one week, affecting more than a half of the patients in the case of plasma Se. No differences were observed for erythrocyte Se when expressed per hemoglobin (*p* > 0.05). In relation to the antioxidant activity, the GPx activity increased significantly (*p* < 0.05) after 7 days of ICU stay.

**Figure 1:**
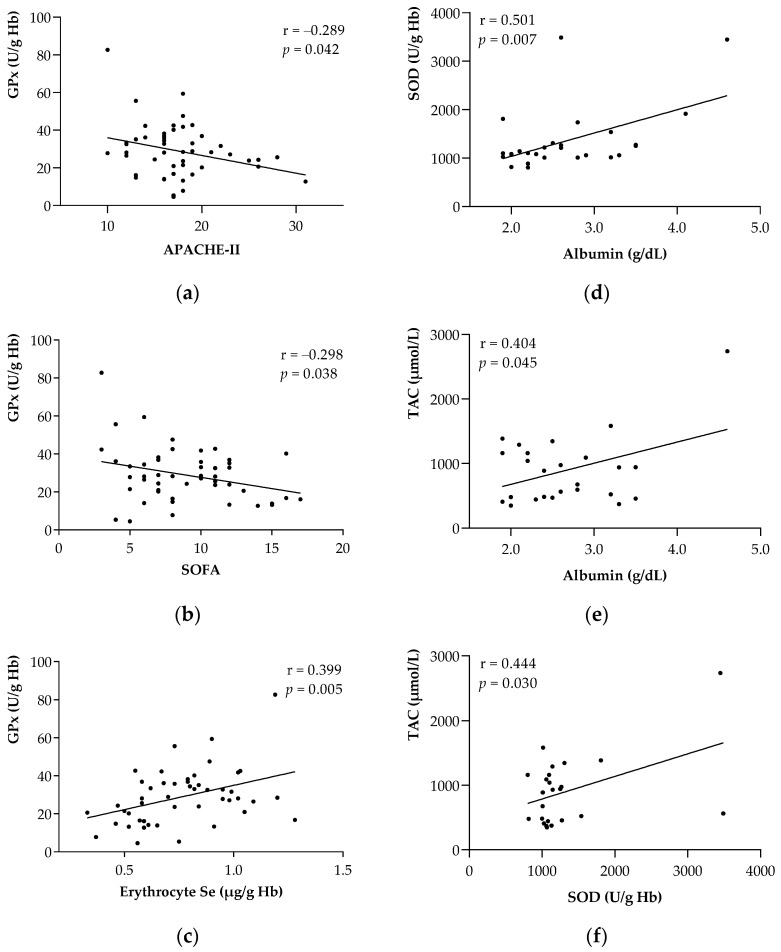
Associations between the antioxidant enzymes and main parameters of the study at the baseline and follow-up. (**a**) GPx with APACHE II at the baseline. (**b**) GPx with SOFA at the baseline. (**c**) GPx with erythrocyte Se at baseline. (**d**) SOD with albumin at follow-up. (**e**) TAC with albumin at follow-up. (**f**) SOD with TAC at follow-up. Abbreviations: APACHE-II = Acute Physiology and Chronic Health Evaluation II; GPx = Glutathione Peroxidase; Hb = Hemoglobin; SOD = Superoxide Dismutase; SOFA = Sequential Organ Failure Assessment; TAC = Total Antioxidant Capacity. Pearson’s correlation coefficient (r) was used to associate all parameters. Statistical significance was considered for *p* < 0.05.

Figure 1 shows a Pearson’s correlation analysis of the antioxidant enzymes with respect to the clinical outcomes, at both the baseline (Figure 1a–c) and follow-up **(**Figure 1d–f). Regarding ICU admission, GPx activity was inversely correlated to severity scores such as APACHE II (r = −0.280, *p* < 0.05; Figure 1a) and SOFA (r = −0.298, *p* < 0.05; Figure 1b) and directly correlated with erythrocyte Se (µg/g Hb) (r = 0.399, *p* < 0.01; Figure 1c). In the case of the significant correlations found at follow-up, albumin was directly correlated to SOD (r = 0.501, *p* < 0.01; Figure 1d) and TAC (r = 0.404, *p* < 0.05; Figure 1e), whereas SOD was directly correlated to TAC (r = 0.444, *p* < 0.05; Figure 1f).

**Figure 2:**
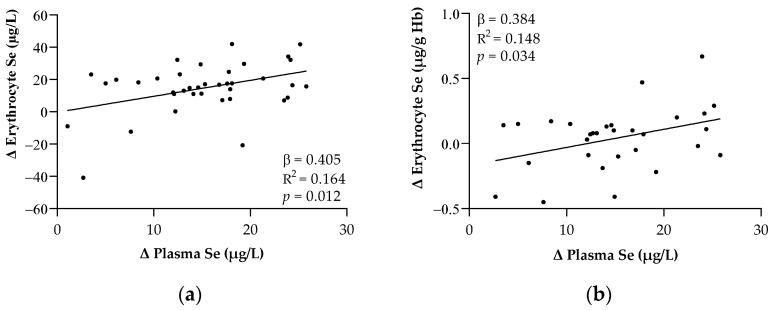
Relationships between the changes in plasma and erythrocyte Se expressed in μg/L (**a**) and μg/g Hb (**b**) after one week of ICU stay. Abbreviations: Hb = Hemoglobin; Se = Selenium. The β (standardized regression coefficient) and *p*-values from single linear regression analyses were obtained. Statistical significance was considered for *p* < 0.05.

Figure 2 shows the significant relationships regarding the changes in the Se levels after 7 days of ICU admission. Changes in the plasma Se levels were associated with changes in the erythrocyte Se levels expressed as both µg/L and µg/g Hb, respectively (*p* < 0.05 in all cases).

**Figure 3:**
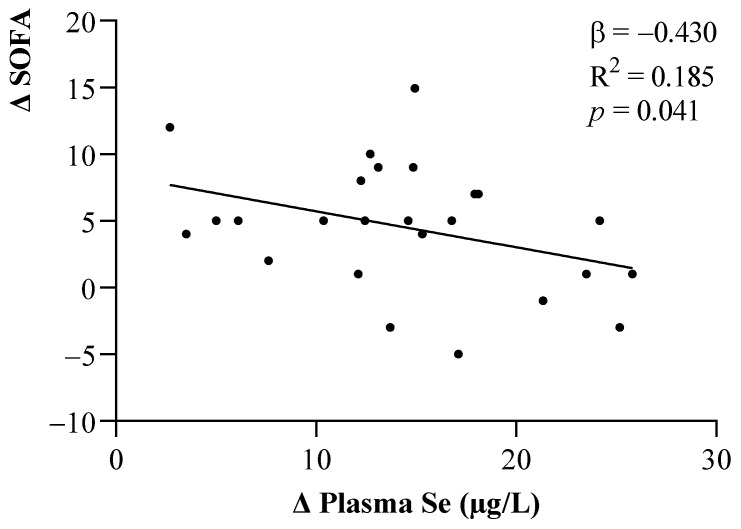
Linear relationship between changes in the SOFA scores and plasma Se levels. The β (standardized regression coefficient) and *p*-values from multiple linear regression analyses were obtained. Statistical significance was considered for *p* < 0.05. Abbreviations: Se = Selenium; SOFA = Sequential Organ Failure Assessment.

The relationships between changes in the SOFA score and plasma Se levels after one week of ICU stay are shown in Figure 3. Smaller changes in the plasma Se levels were seen to be related to greater increases in the SOFA scores (*p* < 0.05).

## 3. Discussion

The present study was carried out to assess the Se and antioxidant levels and their evolution after one week of ICU stay in critically ill patients. Initially, the prevalence of patients showing Se deficiency was 15.4% for plasma Se and 29.2% for erythrocyte Se. Moreover, plasma and erythrocyte Se were seen to be decreased after 7 days of ICU stay, affecting more than half of the patients in the case of plasma Se, despite the standard support received. Contrary to our primary hypothesis, no significant changes in the SOD activity or TAC were observed, though GPx activity increased significantly in this period. Interestingly, GPx activity was inversely related to both the APACHE and the SOFA scores and to erythrocyte Se at the baseline. Counterintuitively, these associations were not found after one week of ICU stay, though decreases in the plasma Se appeared to reflect increases in severity, as assessed by the SOFA score.

It has been reported that most plasma micronutrients decrease as part of the SIRS response in critical patients. This may occur for several reasons (e.g., redistribution processes, increased catabolism, or urinary excretion), making it necessary to interpret the plasma micronutrient concentrations with caution [20,21]. Additionally, both capillary leakage and endothelial dysfunction caused by sepsis or ischemia–reperfusion injury leads to the additional loss of serum selenoproteins into the interstitium [4] or selenoprotein P (SePP) binding to the endothelium [22]. Regarding the Se levels, profoundly lowered plasma Se concentrations have been widely reported at ICU admission [7], but even significantly lower values have been found in patients with SIRS [23]. In our study, the plasma and erythrocyte Se levels (expressed per volume) were lower in ICU patients in comparison with the healthy controls at the baseline—the prevalence of individuals with considerably lowered levels being 15%. This percentage increased by up to four times after 7 days in the ICU. In this line, previous studies have shown that patients who, upon admission, present values < 0.70 μmol/L, which is consistent with the values recorded in our study (0.73 ± 0.14 μmol/L upon admission and 0.54 ± 0.13 μmol/L after 7 days in the ICU), have a 3.5-fold higher mortality rate and a three-fold higher organ failure rate [23].

After one week of ICU stay, the Se levels decreased significantly, elevating Se deficiency and affecting more than 50% of the patients. This decrease in erythrocyte Se levels was not significant when expressed per gram of hemoglobin, however. Although hemoglobin is used to adjust for differences in hematocrit [24], it must be noted that other factors could influence these data, such as the role of hemoglobin in exporting Se from erythrocytes [25] or the observed prevalence of anemia caused by Se deficiency—albeit in animal models [26]. The assessment of the real Se status and its evolution in critical patients presents many difficulties. The main drawback of plasma Se is the interpretation of the results in patients with SIRS [7,9]. Erythrocyte Se seems to be unaffected by SIRS, and it may be a more reliable indicator of the Se status in the critically ill [27]. Nevertheless, erythrocyte results may differ depending on how they are expressed. In this regard, when we examined the changes in plasma Se over time, they were related to the changes in erythrocyte Se, both expressed per volume and per gram of hemoglobin, which corroborates the idea that the risk of Se deficiency in our patients increased during ICU stay.

In reference to Se support, the average amount was less than 40.0 µg of Se per day, which is clearly lower than the Dietary Recommended Values for the healthy adult European population (70.0 µg Se/day) [28] or the suggested Se doses in ICU patients [27]. Our results are consistent with those of other studies, where similar plasma Se levels were found in patients transferred from the ICU to wards [29] and where enteral nutrition did not normalize the plasma levels in the first week of ICU stay [6]. The critically ill patients in our study just received a standard formula with no special supplementation during their ICU stay, and the prevalence of patients with a risk of deficiency increased over time. All this suggests that Se support was insufficient even though the severity scores, and the other clinical parameters improved over time, probably due to the clinical stabilization of the patients. In this regard, Se supplementation remains controversial, mainly due to its narrow therapeutic window [30,31]. Some authors have observed that Se supplementation during 7 days of parenteral nutrition in patients with inflammation increased the Se plasma levels, though not enough to reach or approach the healthy reference values [32]. A recent meta-analysis that evaluated the clinical outcomes of Se supplementation in critically ill patients suggested that it could reduce the overall mortality [33], though the use of high doses could increase the days of ICU stay [34].

Erythrocyte Se has been found to be closely correlated to plasma concentrations and GPx activity in healthy populations [35]. Regarding GPx activity in our critically ill patients, at ICU admission, we observed a direct correlation to the erythrocyte Se levels and an inverse association to the APACHE-II and SOFA scores. It has been previously reported that both decreased serum Se concentration and GPx activity are inversely correlated to clinical outcomes [27]. This fact is in concordance with the observed correlations between the severity scales and GPx activity at the baseline, and with the changes observed in plasma Se. The GPx activity levels increased over follow-up, and the significant correlation to the SOFA score disappeared. This fact could be caused, on the one hand, by the decrease in sample size due to the high patients’ mortality rate during follow-up and, on the other hand, by the decrease in SOFA scores by the hemodynamic stabilization of critical patients, in addition to the underlying processes related to Se metabolism in this deficient situation. It is known that selenomethionine may be incorporated nonspecifically into proteins such as hemoglobin by randomly replacing the (sulfur) methionine [36]. However, knowledge about the redistribution between Se storage and blood is largely lacking, and a decrease in the Se levels does not imply that the selenoenzymes in every compartment are desaturated [19].

On the other hand, in our study, SOD activity and TAC were directly correlated at follow-up and were also associated with low albumin levels. Although we did not clearly observe a significant decrease (*p* = 0.061) in these antioxidant parameters during patient follow-up, other studies have reported decreased antioxidants levels accompanied by lower albumin levels after clinical recovery from severe sepsis [37]. It should be noted that we observed higher TAC in critical patients at the baseline in comparison to healthy controls, maybe due to the initial hypercatabolic state at patients’ arrival in the ICU and their acute-phase response. Nevertheless, plasma TAC must be interpreted with caution due to the heterogeneity of results (i.e., increased plasma TAC has been correlated with worse outcomes after aneurysmal hemorrhage) [38]. In summary, the results of the present study suggest a substantial risk of Se deficiency, with an apparent tendency to further worsen during longer ICU stays, irrespective of the antioxidant and severity parameters, which could affect the overall clinical outcomes in critically ill patients. Furthermore, although Se delivery was ensured in nutritional formulas, it appears insufficient to restore normal values in this population.

Some limitations must be considered in the interpretation of our findings. Follow-up during the 7-days stay in the ICU did not allow us to establish causal relationships, and this study enrolled fewer patients than desired due to the difficulty of obtaining the sample and the inherent variable clinical situation and severity of the patients. On the other hand, there is no information on the other biomarkers of the Se status, such as the levels of the selenoproteins. Therefore, these results should be interpreted with caution, and further studies are needed to assess the impact of long-term Se interventions upon antioxidant activity and the evolution of severity in order to reinforce our findings.

## 4. Materials and Methods

### 4.1. Subjects and Study Design

The present study involved a prospective multicenter analytical design and was carried out of patients from different hospitals in Granada (Spain) (Hospital Virgen de las Nieves, Hospital San Cecilio, Hospital General de Baza, and Hospital Santa Ana de Motril) during the period from March of 2015 to June of 2019. The patients were monitored from admission (baseline) until day 7 of stay (follow-up) in the ICU. Of a total of 65 initially recruited critically ill patients (42% women) by convenience sampling aged 31–77 years (mean age 60 years), 25 (33% women) died during the study period. All eligible participants enrolled in the study were critical patients: (i) aged 18 years or older (ii) with systemic inflammatory response syndrome (SIRS) following SIRS criteria [3] and (iii) who agreed to participate in the study or in which approval of participation was obtained from the family. Exclusion criteria were: (i) refusal to participate in the study as expressed by the patient or his/her legal representatives, (ii) pregnancy, (iii) the presence of highly contagious disease, (iv) allergies, (v) cancer, and (vi) the ingestion of food before obtaining the analytical sample at the baseline. In order to have reference values for the studied parameters and assess the critical condition, control samples were obtained from 56 healthy controls (55% women) from the same region and matched for age, using non-probabilistic consecutive sampling. The subjects in the control group were: (i) adults over 18 years of age (ii) with no use of nutritional supplements, (iii) no disease conditions that could affect their nutritional status, and (iv) individuals agreeing to participate in the study. The present study was conducted in accordance with the principles of the Declaration of Helsinki following the International Conference on Harmonization (ICH)/Good Clinical Practice (GCP) standards [39] and was approved by the Ethics Committee of the University of Granada (Ref. 149/CEIH/2016).

### 4.2. Data Collection

The study data, including age, sex, body mass index (BMI), total proteins, albumin, prealbumin, triglycerides, total cholesterol, hemoglobin, and CRP, were retrieved from the hospital electronic database system and recorded for each study participant at ICU admission (baseline) and after 7 days (follow-up). Biochemical parameters were determined in the Laboratory Analysis Unit of Virgen de las Nieves Hospital (Granada) (ECLIA, Elecsys 2010 and Modular Analytics E170, Roche Diagnostics, Mannheim, Germany). The APACHE-II score and SOFA score were obtained by intensivists in the ICU.

### 4.3. Nutritional Profile

Se intake in the control group was documented through personal interview by staff trained in the use of nutritional techniques, employing questionnaires based on the 24-h recall test [40]. Nutrient intake was then calculated with the Dietowin^®^ application (version 7.1., Barcelona, Spain). The nutritional support protocol (which includes Se support among other micronutrients) in critically ill patients was assessed according to the Clinical Nutrition Units of the hospitals, based on the American Society for Parenteral and Enteral Nutrition and the European Society of Parenteral and Enteral Nutrition guidelines [41]. All patients received standard nutritional support via the enteral, parenteral, or combined routes, administrating nutritional formulas elaborated in the pharmacies of the hospitals or in the form of commercial products. A daily nutritional log was kept for each patient (i.e., type, volume, composition of intake, and tolerance) from admission to 7 days in the ICU. Se support was calculated daily and registered by the nutritionists and was represented as the average of the 7-days period of stay in the ICU.

### 4.4. Blood Sampling and Biochemical Parameters

Two blood extractions of approximately 10 mL were performed (baseline and follow-up) in the morning under fasting conditions. The recorded biochemical parameters were determined using routine hospital analytical assays (ECLIA, Elecsys 2010, and Modular Analytics E170, Roche Diagnostics, Mannheim, Germany). Plasma and cells were separated using 4 mL of blood through centrifugation (4 °C for 15 min at 3000 rpm), and erythrocytes were washed four times with the physiological saline solution. Samples were stored at −80 °C until analytical determination of the different parameters.

### 4.5. Measurement of Se, GPx, and TAC

The determination of both the plasma and erythrocyte Se levels was carried out by inductively coupled plasma mass spectrometry (ICP-MS NexION 300D, Perkin Elmer, Waltham MA, USA) using aqueous solutions from wet-mineralized samples diluted with Triton X-100 and nitric acid. In order to evaluate the prevalence of individuals with a high risk of Se deficiency, the lower range values of plasma and erythrocyte Se (45.6 µg/L and 69.0 µg/L, respectively) of 84 healthy subjects from the same region [42] were taken as the cut-off values.

Commercial kinetic colorimetric methods were used to assess the antioxidant parameters. GPx activity was measured in erythrocyte samples using enzymatic immunological methods for GPx with the Bioxytech GPx-340™ kit (OxisResearch™). Plasma SOD activity was measured using the Randox Ransod kit (Bioxytech^®^ SOD-525™ RANDOX Laboratories Ltd., Antrim, UK). The TAC was assessed in the plasma samples (TAC kit, Jaica, Shizuoka, Japan) [43]. The variability was tested repeatedly with 5 samples and considering variability lower than 5% to be included. The reference values for each antioxidant parameter were determined by the manufacturer, and all parameters were analyzed twice. Se, GPx, and SOD in erythrocytes were all calculated as a ratio to hemoglobin concentration in order to correct for hemodilution processes or inaccuracies associated with the pipetting of packed red blood cells [9].

### 4.6. Statistical Analysis

Qualitative variables were presented as the frequencies (N) and percentages (%). Quantitative variables were expressed as the arithmetic mean ± standard deviation (SD). For continuous variables, the assumption of normality was tested using the Kolmogorov–Smirnov test as a step prior to the application of a parametric or nonparametric model. For the comparative analyses at the baseline and follow-up, they were made of the paired Student *t*-test for parametric samples. For the comparative intergroups analysis, the unpaired Student *t*-test for parametric samples was applied. Correlation analyses and partial correlation coefficients were performed using Pearson’s test to study the relationship between the clinical outcomes and antioxidant enzymes. Likewise, simple linear regression models were used to study the associations between the mean differences of the main outcomes. The SPSS version 26.0 statistical package (IBM SPSS, Armonk, NY, USA) was used throughout. Statistical significance was considered for *p* < 0.05.

## 5. Conclusions

The critically ill patients in our study showed substantially lowered Se levels at ICU admission, and these levels decreased even further after one week of stay in intensive care—a direct correlation observed between the erythrocyte and plasma Se levels, both at the baseline and follow-up. Similarly, GPx activity at the baseline was lower than that observed in healthy controls, being inversely related to the severity scores. In this line, there was an inverse association between the plasma Se levels and the SOFA score, but no relationship between GPx and severity was evidenced after 7 days of ICU stay. Additionally, the standard Se nutritional support in the ICU seemed to be insufficient considering the Dietary Recommended Values and outcomes. Therefore, an adequate increase in the Se supply from the beginning of the ICU stay is advised, aimed at improving both the Se status and the clinical profiles, which could contribute to improving the outcomes and facilitate critical patient recovery.

## Figures and Tables

**Table 1:** Descriptive analysis of the patients at admission to the ICU and the control group, and analysis of the differences between the groups.

Parameters	Healthy Controls Mean (SD) *N* = 56	ICU Patients Mean (SD) *N* = 65	*p*-Value
BMI (kg/m^2^)	25.0 (3.26)	26.7 (4.77)	0.069
Patient 7-day mortality (n/N, %)	-	25/65 (38.5)	-
APACHE-II score	-	17.2 (4.94)	-
SOFA score	-	9.34 (3.90)	-
Total proteins (g/dL)	7.39 (0.37)	5.19 (0.84)	0.001
Albumin (g/dL)	4.41 (0.27)	2.73 (0.62)	0.001
Prealbumin (mg/dL)	27.9 (5.10)	12.7 (6.72)	0.001
Triglycerides (mg/dL)	110.7 (58.1)	186.8 (124.0)	0.001
Total cholesterol (mg/dL)	206.0 (40.8)	108.9 (38.1)	0.001
Hemoglobin (g/dL)	13.4 (1.88)	10.9 (2.70)	0.001
CRP (mg/L)	0.22 (0.25)	19.9 (13.1)	0.001
Se support (µg/day)	69.4 (30.3)	37.8 (23.1)	0.001
Plasma Se (µg/L)	76.4 (18.1)	56.2 (12.9)	0.001
Erythrocyte Se (µg/L)	109.3 (22.5)	79.7 (14.9)	0.001
Erythrocyte Se (µg/g Hb)	0.84 (0.24)	0.76 (0.22)	0.098
GPx activity (U/g Hb)	42.2 (28.7)	29.0 (14.3)	0.004
SOD activity (U/g Hb)	1628.1 (1439.0)	1501.1 (551.1)	0.546
TAC (µmol/L)	801.5 (374.7)	1100.6 (490.5)	0.001

Data are expressed as the mean ± standard deviation. Abbreviations: SD = Standard Deviation; APACHE-II = Acute Physiology and Chronic Health Evaluation II; BMI = Body Mass Index; CRP = C-Reactive Protein; GPx = Glutathione Peroxidase; Se = Selenium; SOD = Superoxide Dismutase; SOFA = Sequential Organ Failure Assessment; TAC = Total Antioxidant Capacity. For the comparative analysis between groups, the unpaired Student’s *t*-test for parametric samples was used. Statistical significance was considered for *p* < 0.05.

**Table 2:** Comparative analysis of the studied parameters at baseline and follow-up.

Parameters	Baseline Mean (SD) *N* = 65	Follow-Up Mean (SD) *N* = 40	ΔChange (%)	*p*-Value
SOFA score	9.04 (3.39)	5.12 (3.50)	−43.4	0.001
Albumin (g/dL)	2.85 (0.58)	2.63 (0.67)	−7.71	0.139
Prealbumin (mg/dL)	12.5 (4.65)	16.3 (9.80)	30.4	0.143
CRP (mg/L)	19.8 (11.8)	10.7 (8.63)	−46.0	0.001
Plasma Se (µg/L)	57.8 (11.6)	42.6 (10.9)	−26.3	0.001
Erythrocyte Se (µg/L)	84.0 (15.4)	69.2 (14.9)	−17.6	0.001
Erythrocyte Se (µg/g Hb)	0.80 (0.24)	0.76 (0.20)	−5.00	0.343
GPx activity (U/g Hb)	30.6 (18.6)	47.3 (27.9)	54.6	0.016
SOD activity (U/g Hb)	1528.6 (587.2)	1377.2 (652.2)	−9.90	0.265
TAC (µmol/L)	1016.3 (445.1)	849.8 (509.4)	−16.4	0.061
**Selenium adequacy**				
Plasma Se < 45.6 µg/L (n,%)	10 (15.4)	25 (62.5)	150.0	0.033
Erythrocyte Se < 69.0 µg/L (n,%)	19 (29.2)	20 (50.0)	5.26	0.167

Data are expressed as the mean ± standard deviation. Abbreviations: SD = Standard Deviation; GPx = Glutathione Peroxidase; Hb = Hemoglobin; Se = Selenium; SOD = Superoxide Dismutase; SOFA = Sequential Organ Failure Assessment; TAC = Total Antioxidant Capacity. Δ Change (%) = Percentage change from baseline to follow-up. For the comparative analysis between groups, the paired Student’s *t*-test for parametric samples was used. Statistical significance was considered for *p* < 0.05.

## Data Availability

Data available on request due to restrictions on privacy. The data presented in this study are available on request from the corresponding authors [E.P. and J.M.-L.].
